# Telemedicine Implementation in COVID-19 ICU: Balancing Physical and Virtual Forms of Visibility

**DOI:** 10.1177/19375867211009225

**Published:** 2021-06-02

**Authors:** Nirit Putievsky Pilosof, Michael Barrett, Eivor Oborn, Galia Barkai, Itai M. Pessach, Eyal Zimlichman

**Affiliations:** 1 Cambridge Digital Innovation—CJBS & Hughes Hall, 2152 University of Cambridge, United Kingdom; 2 Cambridge Judge Business School (CJBS), 2152 University of Cambridge, United Kingdom; 3 Warwick Business School, University of Warwick, United Kingdom; 4 Sheba BEYOND, 26744 Sheba Medical Center, Tel Hashomer, Israel; 5 26744 Sheba Medical Center, Tel Hashomer, Israel; 6 Sackler Faculty of Medicine, Tel-Aviv University, Israel; 7 Sheba’s Talpiot Medical Leadership Program, Israel

**Keywords:** model of care, inpatient telemedicine, visibility, healthcare design, control room, COVID-19

## Abstract

**Objective:**

This case study examines the implementation of inpatient telemedicine in COVID-19 intensive care units (ICUs) and explores the impact of shifting forms of visibility on the management of the unit, staff collaboration, and patient care.

**Background:**

The COVID-19 crisis drove healthcare institutions to rapidly develop new models of care based on integrating digital technologies for remote care with transformations in the hospital-built environment. The Sheba Medical Center in Israel created COVID-19 ICUs in an underground structure with an open-ward layout and telemedicine control rooms to remotely supervise, communicate, and support the operations in the contaminated zones. One unit had a physical visual connection between the control room and the contaminated zone through a window, while the other had only a virtual connection with digital technologies.

**Methods:**

The findings are based on semistructured interviews with Sheba medical staff, telemedicine companies, and the architectural design team and observations at the COVID-19 units during March–August 2020.

**Results:**

The case study illustrates the implications of virtual and physical visibility on the management of the unit, staff collaboration, and patient care. It demonstrates the correlations between patterns of visibility and the users’ sense of control, orientation in space, teamwork, safety, quality of care, and well-being.

**Conclusions:**

The case study demonstrates the limitations of current telemedicine technologies that were not designed for inpatient care to account for the spatial perception of the unit and the dynamic use of the space. It presents the potential of a hybrid model that balances virtual and physical forms of visibility and suggests directions for future research and development of inpatient telemedicine.

The response to the COVID-19 pandemic has globally transformed hospital operations. The challenge and threat of an imminent surge of COVID-19 patients overloading hospitals drove healthcare institutions to rapidly change their physical built environment, implement digital technologies, and develop new models of care. The main logic behind the transformations was the need to increase hospital capacity, provide the best medical treatment for the COVID-19 patients, and protect the medical personnel from infection.

One of the primary outcomes of the healthcare system’s response to the COVID-19 pandemic was the acceleration of telemedicine technologies, which has evolved, as some predicted, from a technology-driven novelty to an essential component of healthcare delivery in most medical specialties and fields (Wilson & Maeder, 2015; Zimlichman, 2005). In addition to the development of telemedicine programs for outpatient care, hospitals recognized the potential of implementing telemedicine for inpatient care to reduce direct contact with COVID-19 patients (Dhala et al., 2020; Igra et al., 2020; Vilendrer et al., 2020). The new model of care in COVID-19 ICU evolved from existing models of electronic intensive care unit (e-ICU), developed in the United States to allow nurses and physicians to remotely monitor the status of many patients in ICUs in multiple hospitals (Hollander & Carr, 2020). Inpatient telemedicine in ICUs demonstrated its potential to enhance care (Huffenberger et al., 2019; Khunlertkit & Carayon, 2013) and its feasibility across diverse settings as a response to the COVID-19 pandemic (Vilendrer et al., 2020). It was also found to support isolated patients in communication with family and enhance the patient and family experience in the critical care setting (Huffenberger et al., 2019; Voo et al., 2020). Yet, hospitals and providers face important choices and challenges related to models of care, technological design, staff roles, regulatory, and financial and legal issues (Koenig, 2019; Lazzara et al., 2015).

The new model of care by inpatient telemedicine transformed one of the most significant aspects of inpatient care, the visibility of patients and staff within the unit. The shift from physical face-to-face communication to virtual online synchronous communication of the ICU’s users, acting within the built environment of the unit. has important implications for healthcare services and hospital design. Yet, previous research on visibility focused on the physical visibility within hospital units and its impact on healthcare outcomes, or on virtual visibility for telemedicine communication with patients at home, and lacks consideration of integrating physical and virtual forms of visibility for inpatient care that has become more common due to the COVID-19 crisis.

***The new model of care by inpatient telemedicine transformed one of the most significant aspects of inpatient care, the visibility of patients and staff within the unit. The shift from physical face-to-face communication to virtual online synchronous communication of the ICU’s users, acting within the built environment of the unit. has important implications for healthcare services and hospital design.***

**Physical visibility**, by which we mean the direct line of visibility via human eyesight, is a major issue in the design of healthcare services. There is a strong body of research documenting the importance of visibility in healthcare settings, demonstrating how visibility enables or prohibits healthcare professionals’ ability to monitor, control, or manage situations. There is evidence that visibility has a significant impact on patient safety, including patient fall rates and mortality rates (Leaf et al., 2010; Lu et al., 2014), and on the performance of healthcare professionals, including situational awareness, communication, and teamwork (Lim et al., 2020; Lim & Zimring, 2020; Peavey & Cai, 2018). Previous research also demonstrates the association between architectural design layout of ICUs and nurse-to-patient visibility parameters as well as the important effects on patient observation and monitoring opportunities (Hadi & Zimring, 2016; Pettit et al., 2014). Higher visibility levels are also associated with higher collaborative communication among nurses and physicians (Gharaveis et al., 2020). A review of the literature on decentralized inpatient unit design, with physically dispersed staff spaces (Peavey & Cai, 2018), showed links to reduced teamwork (Parker et al., 2012; Pati et al., 2015; Real et al., 2017), task support (Nanda et al., 2015), social support (Real et al., 2017; Zborowsky et al., 2010), and to increased feelings of isolation (Nanda et al., 2015; Pati et al., 2015). Visibility of patients and among peers is especially critical in high-stress, high-acuity situations, as in the ICU, when nonverbal cues become increasingly essential (Grimes et al., 2017; Pati et al., 2015).

**Virtual visibility**, which refers to visibility mediated by digital technologies, is often associated with telemedicine in outpatient care. A review on the key aspects to consider when designing telemedicine spaces at the hospital or a clinic addressed the size and location of the room, lighting, interior surfaces for background, and site identification (The Center for Health Design, 2020). Research on camera placement for videoconferencing shows a significant impact of distance from the camera and eye gaze angles on the outcome of telemedicine consultations and user satisfaction (Tam et al., 2007). The Facility Guidelines Institute at the United States highlights that design of spaces used for telemedicine communications should strive to provide what would be expected for the same communication taking place in person, including patients’ privacy, safety, quality of care, and patient experience (Taylor, 2020). Previous research also argues that telemedicine is not about technology but about people. It suggests adopting a user-centered design methodology for the design and development of telemedicine systems to support the quality of care (Martínez-Alcalá et al., 2013). Research conducted before the COVID-19 crisis also argues that digital communication is often viewed as ineffective at connecting team members in critical care (Pati et al., 2015; Peavey & Cai, 2018). As such, examining the manner in which the sudden implementation of telemedicine technologies in ICUs during COVID-19 is important to explore.

## Method

This case study examines the implementation of inpatient telemedicine in COVID-19 ICUs and questions what is the impact of shifting forms of physical and virtual visibility on the hospital healthcare services. The case study analyzes the dynamic interrelationship between the architectural design of the COVID-19 units, digital technologies, and healthcare services. The exploratory case study involving interpretive qualitative inquiry analyzes the affordances of the digital technologies and physical materiality to support different patterns of visibility and examines the stakeholders’ interpretive understanding of the visibility impact on the management of the unit, staff teamwork and collaboration, and patient care. By physical materiality, we are referring to the physical properties of an artifact such as window, club car, and personal protection equipment (PPE) and which are distinct from digital materiality which are increasingly used to denote the material properties of digital artifacts such as software programs.

The case study focuses on the Sheba Medical Center (MC) at Tel HaShomer, a 1,900-bed tertiary hospital in Israel. Supported by the ARC Innovation Center at Sheba MC, the hospital rapidly developed a new model of care for ICUs with inpatient telemedicine technologies during the COVID-19 crisis. The case study received approval from the institutional review board at the hospital as part of a broader research project studying the strategic development and planning of smart hospitals. The first author obtained access and collected data from the sources outlined in Online Appendix 1 in collaboration with co-authors from the hospital.

The case study, conducted in March–August 2020 during the first and the second waves of COVID-19 in Israel, is based on 30 formal interviews with Sheba medical staff, telemedicine companies, and the architectural design team; 2 days of observations at the COVID-19 units; and guided tours of the COVID-19 units by the Sheba MC director of the COVID-19 division and the Sheba MC director of infrastructure and building. The formal one-on-one interviews were semistructured, including topics such as the use of the telemedicine technologies, the impact of the built environment, and the change in professional practice. The volunteer subjects for the interview were selected purposefully by consultation with the hospital management and according to work availability. The participants included 11 physicians, three nurses, three IT administrators, two human experience directors, five start-up directors, and six architects and engineers. Each interview took approximately 30–60 min, most were held in person in the hospital and some were conducted virtually by zoom due to curfew restrictions. Most of the interviews were recorded in Hebrew (with the consent of the interviewees), transcribed, and professionally translated into English under supervision of the researchers.

The 2 days of field observations of the COVID-19 ICU focused on investigating the visibility from the control room and its outcomes on the units’ operations. Field observations and interview notes were recorded for analysis. Additional data included media coverage, hospital webinars, and analysis of the architectural plans (Online Appendix 1). The case study was presented to the hospital board of directors for their information and comments.

The thematic qualitative data analysis, based on principles of naturalistic inquiry (Lincoln & Guba, 1985) and a grounded approach to conceptual development (Golden-Biddle & Locke, 2007), was adopted to identify emerging themes from the interviews and observations. Related and similar ideas were clustered together through the coding of interviews and field notes eliciting supporting quotes as evidence for the case analysis. The emerging themes were characterized by their impact on the management of the unit, teamwork and collaboration, and patient care. Table 1 provides more details of these themes and their associated subthemes (Table 1).

**Table 1.** The Impact of the Shifting Forms of Visibility on the Healthcare Services at the Sheba Medical Center COVID-19 ICU.

Healthcare Services	Impact on the Healthcare Services	Examples of the Shifting Forms of Visibility
Management of the unit	Orientation and spatial perception	Segregated views on screens of monitoring devices and video cameras
	Supervision of staff	Robot to provide dynamic and personal communication
	Control of operations	Club-car to drive through the beds an overview of the whole unit
Teamwork and collaboration	Professional processes	Video cameras and robot supported change in processes
	Communication practices	The window provided direct observation and low-tech methods of communication
	Staff hierarchies	PPE caused loss of identity and limitations in staff work
Patient care	Safety and quality	Video cameras and robot were used for infection control
	Patient privacy	Privacy curtains were not used because of the video cameras
	Family support	Virtual communication was not sufficient for families and patients

## The New Model of Care at the Sheba MC in Israel

**Figure 1.**
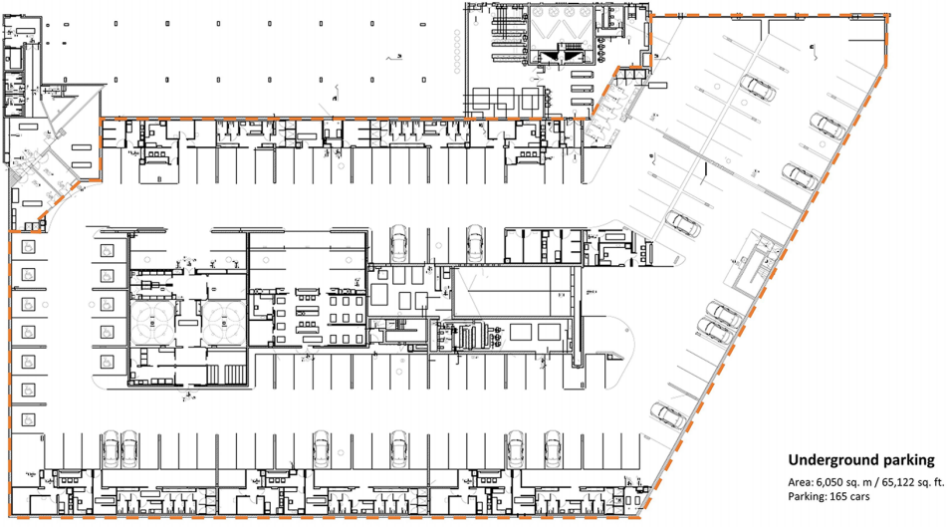
Architectural plan of the underground parking lot at Sheba MC.

**Figure 2.**
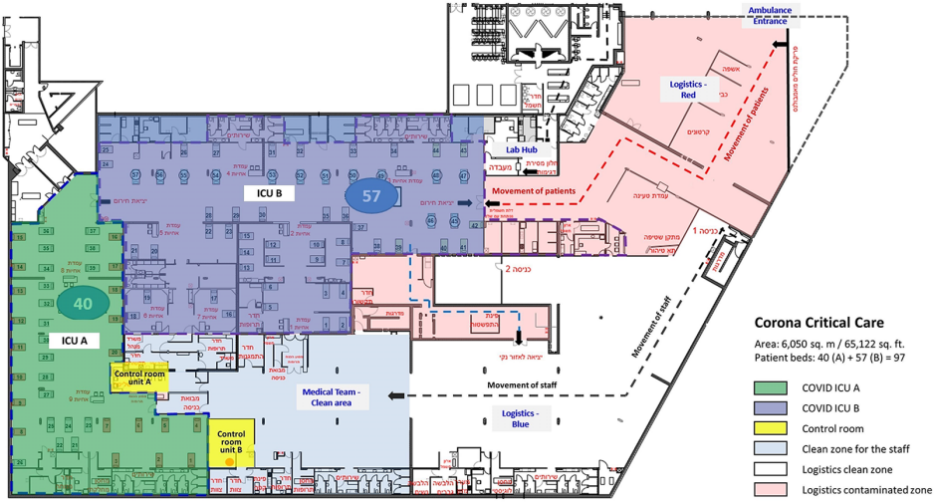
Architectural plan of the COVID-19 critical care hospital at Sheba MC.

In response to the COVID-19 crisis, Sheba MC constructed rapid design solutions to increase patient bed capacity and to maintain regular hospital operations. Sheba MC added 500 beds to its existing total of 1,900 beds in the hospital (+26%), including 97 ICU beds in two underground COVID-19 critical care units (Leshem et al., 2020). The isolated COVID-19 ICUs were constructed in only a few days in an underground parking lot, originally designed to serve as a fortified emergency hospital for non-ICU-level patients in times of war (Figure 1). The main objective behind the decision to locate the COVID-19 ICUs in the underground parking was the need to isolate the COVID-19 patients from the rest of the operating hospital units and to save money and time by utilizing an existing structure. The large site of the parking lot of 6,050 sq. m (65,122 sq. ft) was divided into two ICUs with separation of clean zones, contaminated zones, and designated circulation routes (Figure 2). Clean zones were completely separated from contaminated zones, using double-door vestibules for donning and doffing of PPE and split air systems. The site was equipped with special infrastructure for electricity, medical devices, and oxygen lines.

The two ICUs were designed with a special control room in the clean zone with telemedicine devices to remotely supervise, communicate, and control the operations in the contaminated zone. Unit A with an area of 1,100 sq. m. (11,840 sq. ft.) has an L shape open space for the contaminated zone and an adjacent clean zone control room with sealed glass windows between the zones. While there was no direct passage from the clean control room to the contaminated zone, the adjacent location provided a visual sight between the two zones. Unit B has an area of 1,700 sq. m. (18,300 sq. ft) with a square shape and is divided into six subzones for operation by the level of care. The control room of Unit B is located in the clean zone at a distance from the unit’s contaminated zone with no direct physical sight of the clinical area of the unit (Figure 2).

Sheba MC, through its ARC innovation Center, employed different technologies to support remote patient care. The objective was to upscale ICU coverage, reduce staff infection risk, and lessen errors related to working in protective gear through constant audiovisual communication between the clean control room and the contaminated zone teams. The technologies for monitoring, physical examination, management, and audiovisual communication included a video camera on each patient, spatial video cameras, and mobile InTouch Telepresence robots. The medical staff worked in two teams: one team in the contaminated zone with PPE and the second team supporting them from the clean zone control room using remote telemedicine technologies. The objective was to have minimal staffing inside the contaminated zone to reduce infection exposure of the staff, while recognizing the necessary bedside intensive care of nurses, physicians, and support personnel for critically ill patients. The divided teams, therefore, worked 12-hr shifts, 3 hr in the contaminated zone and 3 hr in the clean zone in rounds.

## The Impact of the Shifting Forms of Visibility

The new model of care at the Sheba MC COVID-19 ICUs generated new forms of visibility enabled through digital technologies and physical materiality on the management of the unit, the teamwork and collaboration of the staff, and the care for the patients. Table 1 displays three key themes for each category including a description of each theme.

### Management of the Unit

The COVID-19 ICUs were operated from the control room by the director of the unit, leading physicians, and head nurse. They were responsible for deciding on patient care and staff operation remotely. The shifting forms of visibility created challenges in orientation and spatial perception, supervision of staff, and control of operations.

#### Orientation and spatial perception

The screens in the control room, presenting the monitoring devices and the video cameras of the patients in the contaminated zone, created a segregated view lacking a holistic conception and spatial integration of the whole unit.

One of the things that the camera does is it divides the whole into parts. In the ICU, there is a concept of control of space. To operate the unit, you need to understand who is next to which bed, where the equipment is, where something is happening. (Medical director)

The multiple cameras provided cumulative screenshots so that many foci of patient beds could be monitored. However, the foregrounding of the foci made the relationships between them obscured.

#### Supervision of staff

**Figure 3.**
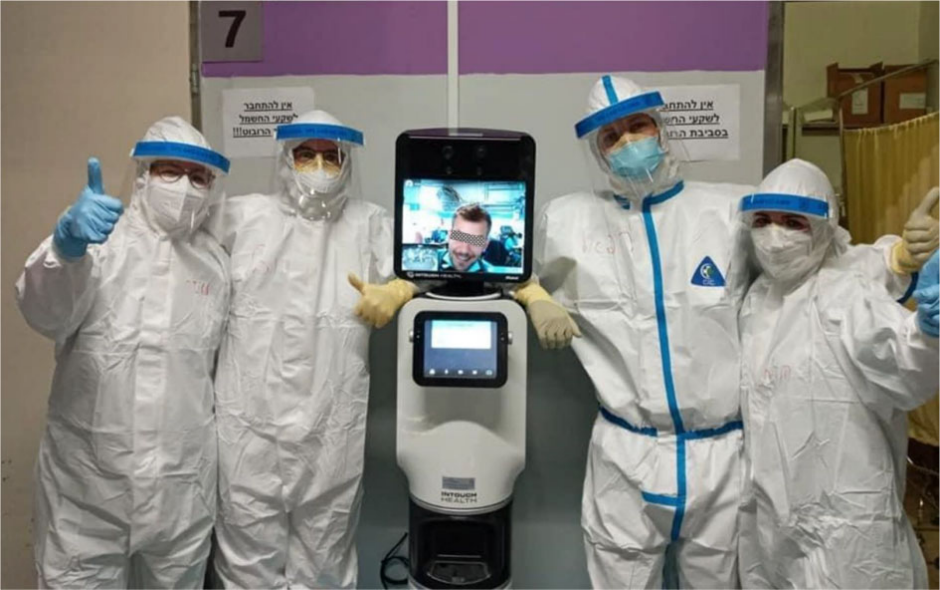
The InTouch Telepresence robot depicting a recognizable human face with staff members covered by personal protection equipment. *Source*: Sheba MC (April 2020).

The director and specialists supervised the resident doctors and nurses working in the contaminated zone from the control room. The audiovisual technologies afforded medics and nurses to share their expertise without the discomfort of PPE. The InTouch Telepresence robot, imitating human eye-level sight, supported the management of the unit by providing a dynamic view of the unit and a more personal virtual face-to-face communication with the staff (Figure 3).

The robot was the most efficient piece of equipment. On the one hand, the ability to see from more than one angle, and on the other hand, the ability of the person on the other side to see the face of the person who was speaking to them. That was a game changer. It is as if you are in the room. You can move from place to place, to focus, and to distance. (Medical director)

The ability to combine the affordances of a dynamic and controllable camera on the robot to augment the fixed views of the video cameras was significant. This highlights the need to have a combination of options to adequately supervise activities, since robots are not fixed to one place (the way a patient bed might be). For supervision, interrelationships between the dynamically changing units are an important factor.

#### Control of operations

**Figure 4.**
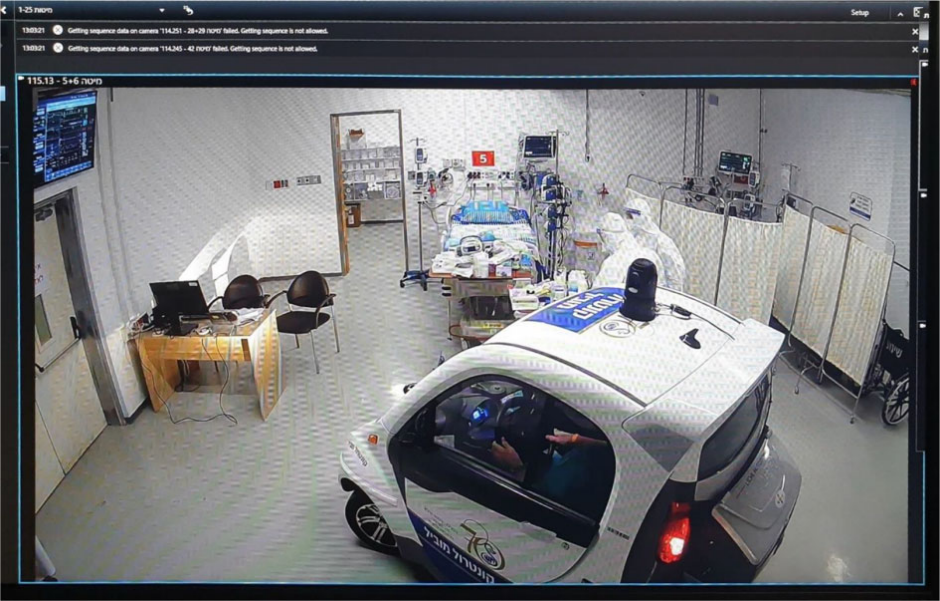
A camera screen showing the club-car driving through the contaminated zone for close-up and dynamic observation. *Source*: Sheba MC (April 2020).

To further enhance control of operations and accommodate the limitations of remote fixed camera views and deficiency of robots, the hospital applied an innovative solution for the directors to enter the contaminated zone without putting on PPE by driving a closed club car in the unit (Figure 4). The club car provided the directors with a sense of control over the unit by enhancing their ability to maneuver and make visible the patients, staff, and unit as a whole. The club car added dynamic control and also the ability to get close, though was reserved only for the directors as limited numbers could fit into car and benefit from this view.

### Teamwork and Collaboration

The division of the staff into two groups, one working in the contaminated zone with PPE and the other supporting them from the control room in the clean zone, increased the need for teamwork and collaboration. The shifting forms of visibility impacted professional processes and changed staff’s communication practices and staff’s hierarchies.

#### Professional processes

The need to take care of patients remotely, staying away from bedside where possible and watching from a distance led the staff to engage with patient monitoring in new ways. The different visibility affordances of the two units led to different work processes by the physicians and nurses. For example, in Unit A, the nurses did bedside documentation for every patient supported by their visual team member through the window, while in Unit B, the lack of the window caused them to develop a method for remote documentation supported by digital telemedicine. Their dependency on virtual visibility led to the decision that when they see a need, they could quickly get dressed with the PPE and enter the unit.

#### Communication practices

**Figure 5.**
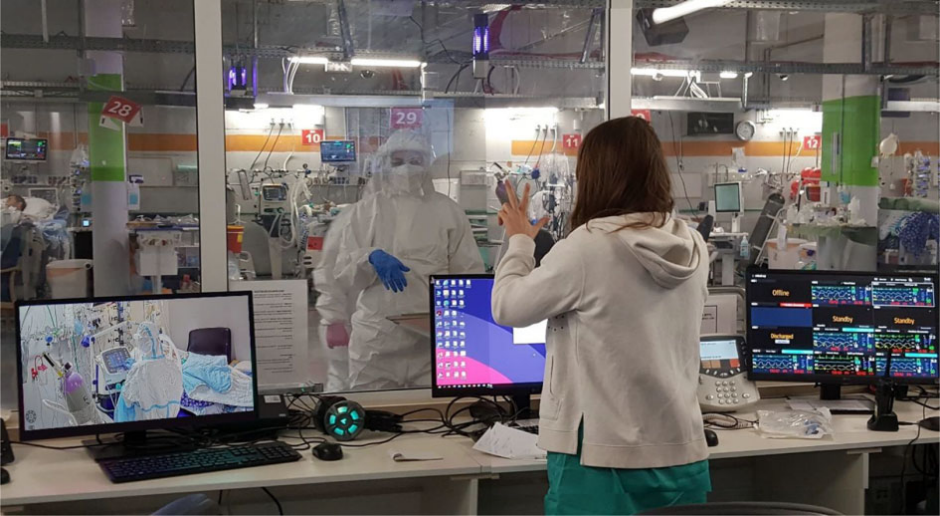
Collaboration by face-to-face communication through the window between the control room and the contaminated zone in COVID Intensive Care Unit A. *Source*: The author (August 2020).

Unit A, with the window between the control room and the clinical contaminated zone, provided direct observation and face-to-face communication, overcoming the acoustic barrier by low-tech solutions such as hand gestures and hand-written notes (Figure 5). Without the physical visibility through the window, the staff in Unit B used the cameras and the robot much more than the staff in Unit A.

The communication and control in Unit A with the window were much simpler. You could see the patients and the caregivers with your own eyes, and you could also use the telemedicine devices, so it was much more comfortable. Also, the ability of staff inside to come to the window and show you: This is the medicine, this is what came out, this is the situation…is much more efficient. It can indeed all be done by video, so it is not a necessity but an advantage. (Medical director)

The case shows the potential to support shifting communication patterns by creatively augmenting analog forms of communication with telemedicine technologies.

#### Staff hierarchies

The hierarchies between physicians and nurses and experienced physicians and residents shifted.

The PPE covered any characteristics of the professional role. When help was needed, any pair of free hands came to the rescue. It did not matter if it was a doctor or a nurse (Head nurse).

Usually, the residents speak with the families, but in the COVID-19 ICU, when families were not allowed to visit, it was the senior doctors who communicated with them every day to provide the most detailed and accurate information (Medical director).

Visibility by camera also created a neutral situation where no one is more privileged than another screenshot. Opposite to human vision that tends to focus on certain persons more because we privilege them as more important, the camera is agnostic (Mengis et al., 2018).

### Patient Care

The use of telemedicine technologies to remotely monitor and supervise patient care transformed the concept of inpatient care. The shifting forms of visibility impacted patient safety and quality, patient privacy, and family support.

#### Patient safety and quality

The new model of care brought up questions of the important role of personal connection between the physician and the patient.

In virtual care, I do not feel that I’m giving my patients what I’m supposed to be giving them as a caregiver. And that does not matter how much we improve the technology if you cannot place your hand on the patient when you give him bad news…but on the other hand, with remote technologies, I can be there for him even when I’m not physically present. (Medical director)

Virtual visibility required staff to develop new ways of showing empathy and making a human connection. The impact of the shifting forms of visibility on the patient’s experience was expressed by one of the patients who was hospitalized for an extended period in the COVID-19 ICU and came to visit the unit a few weeks after he was discharged. When asked what he remembered from his experience in the COVID-19 ICU, he said, “I don’t remember anything other than the stewardess on skates…” referring to the robot moving on wheels.

Why did the patient remember the robot and he didn’t remember anyone else? Everyone else was people without faces. Everyone who wasn’t the robot had a mask and their face was covered, looking like astronauts… (medical director).

For the patient, the sense of human empathy was given through a visible face, without mask and PPE protection, even though digitally mediated (Figure 3).

The virtual visibility enabled through telemedicine technologies enhanced patient safety and quality of care by supervising the behavior of the staff to prevent cross-infection from patient to patient.

I was using the robot to wander around the room, going from bed to bed, and all of a sudden, I saw a doctor lift his hand, and the entire seam of his robe was torn…. He didn’t notice. No one noticed. This was thanks to the robot. In a normal situation, when I do my rounds surrounded by five to six doctors, I would not have noticed a tear in a doctor’s robe, I would be looking at the patient, not at the doctors. (Medical director)

#### Patient privacy

The use of telemedicine technologies, including the video cameras per patient in the contaminated zone, denied patient privacy. The hospital provided portable curtains on wheels but they created a barrier for virtual visibility and were moved away. The lack of visual privacy for the patients and the staff aroused significant ethical issues addressed in hospital policies.

There was no privacy for the patients. There weren’t even curtains between the beds because of the cameras. In this situation, the patients who weren’t ventilated witnessed the disease’s natural progression…. They could see, maybe only a few meters from them, what they were going to look like in another 3–4 days. That was horrible! (Resident doctor)

#### Family support

The visibility affordances also transformed models of family support. Although the hospital provided the families with a special facility in the hospital for remote virtual communication with their patients and the staff in the ICU, “digital visiting hours,” they soon realized virtual visibility was not sufficient both for the families and for the patients.

The family meeting with the patient can be virtual—they can speak from home—but they want physical contact. Telemedicine, as good as it is, is not enough. Many people need physical contact with the patient to be close to their loved one. They are willing to put on the PPE to go into the unit even if they might risk their health. (Hospital director)

The team recognized that even ventilated patients were aware of family presence and got emotional. “One patient shed a tear, and the blood pressure and pulse of another went up” (medical director). However, bringing family physically onto the ward was not always possible and limited the numbers who could visit. Specific focused visibility of loved ones at punctuated points of the day through cameras could mediate the present providing compassion for the family, and encouraging the patient, without the need for PPE masks covering faces.

The location of the COVID-19 ICUs in the underground site, with no visibility of the outdoors, natural sight, and daylight, impacted the well-being of the patients and staff.

I think that the underground location was a very bad choice, it was like working in a submarine…. I think people developed PTSD because of that place. From the place itself and the general situation. You cannot separate the two. I’m certain that if we were in a regular department with windows, it would have made a difference. (Medical director)

Many staff members noted that they believe the underground location also caused depression to the patients.

When they were coming out of an induced coma, and we wanted to stop the anesthetics, the patients had very bad responses. If they were tested negative to COVID-19 and transferred to the regular ICU, they were able to wake up in a day. We couldn’t accomplish that in a week, even more. (Medical director)

## Discussion

The new model of inpatient telemedicine care in the COVID-19 ICUs generated new forms of visibility by digital technologies and physical materiality. The insights of the case study demonstrate the relationship between the affordances of the virtual and physical patterns of visibility and their impact on the management of the unit, teamwork and collaboration, and patient care. For the management of the unit, challenged by remote control of operations, the limitations of the video cameras that provided specific direction, focus on seeing, static, and high angle of view were balanced by the robot with dynamic distance eye-level view and the addition of a club car with dynamic multi-focus specific and neutral direction. The teamwork and collaboration of the staff, challenged by the use of PPE, was enhanced by the window between the control room and the clinical zone that afforded a diverse relationship, specific and neutral direction, and the robot with multi-focus personal relationship. Patient care, challenged by the lack of privacy and family support, was supported by the visibility affordances of the video cameras and robot for a specific relationship, specific direction, and change of distance that enhanced the patient safety and level of care.

The case study illustrates the affordances and limitations of the digital technologies and physical materiality used in developing the new model of care. The case study demonstrated the limitations of current digital telemedicine technologies that were not designed for inpatient care to account for the spatial perception of the unit and the dynamic use of the space. Augmenting a diverse range of visibilities was crucial for orientation and control of the unit, significantly important for the management of the unit responsible for allocating staff and resources, and for the teamwork and collaboration among the staff. Similar to other studies (Khurrum et al., 2020; Subramanian et al., 2020), this case study also shows the challenge of using multiple digital technologies for patient monitoring and visual communication on segregated screens and the need to develop one comprehensive platform to support the dynamic multipurpose use of the technologies.

***The case study demonstrated the limitations of current digital telemedicine technologies that were not designed for inpatient care to account for the spatial perception of the unit and the dynamic use of the space.***

To overcome the limitations of the telemedicine technologies that were not specifically designed to support the complex and stressful (Shen et al., 2020), high-touch and multi-person care process in the ICU (Leaf et al., 2010; Lu et al., 2014), the hospital skillfully augmented with other forms of physical visibility, including the architectural design of the window between the control room and the contaminated zone and the club car drive. The advantages of the InTouch Telepresence robot for management, patient care, and family support demonstrated the need for visibility patterns that mimic human sight. The case study provides important insights on the use of digital technologies and physical materiality to support telemedicine for inpatient care which should be designed for infection control, foster family support, and whose robotic capabilities can offer human augmentation.

***The case study provides important insights on the use of digital technologies and physical materiality to support telemedicine for inpatient care which should be designed for infection control, foster family support, and whose robotic capabilities can offer human augmentation.***

The combination of virtual and physical forms of visibility, as in the design of the control room in unit A with the window, demonstrated the advantages of providing diverse patterns of visibility to enhance different uses. Physical visibility based on the user’s dynamic perception while present in space, on-site, and virtual visibility that provides control over a distance of view by zooming in and out, movement in space by robots, and angle of view for an overview or eye-level sight was significant in maintaining the essential characteristics of an ICU: close supervision on patients, collaborative teamwork, and moral responsibility for family members.

## Conclusions

The hospital’s resilience was enhanced by the implementation of digital technologies for remote care and the design of the built environment to support new models of care by inpatient telemedicine. One of the main implications of the rapid transformations in healthcare services, demonstrated in this case study, is the need for a breadth and multiple forms of visibility. The move from on-site physical visibility to remote virtual visibility by digital technologies has a significant impact on the dynamics of care provision of inpatient units. Different from outpatient care, where virtual visibility is mainly a means for enhanced communication between the caregiver and the patient, in inpatient care, and specifically in ICU, virtual visibility needs to support different purposes such as management, teamwork, collaboration, and care, relating to different users, including hospital directors, physicians, nurses, technicians, families, and patients, in changing conditions over time.

The complexity of the hospital operations and the often-conflicting needs of different end users demand a holistic approach to inpatient care visibility. The case study presents the potential of a hybrid model of virtual and physical forms of visibility that was developed to overcome the limitations of current telemedicine technologies that were not designed for hospital care. The diverse patterns of visibility by digital technologies and physical materiality supported the healthcare services of the unit by enhancing control, perception of the unit as a whole, orientation in space, safety, infection control, collaboration, and well-being of all users.

***The diverse patterns of visibility by digital technologies and physical materiality supported the healthcare services of the unit by enhancing control, perception of the unit as a whole, orientation in space, safety, infection control, collaboration, and well-being of all users.***

Future models of remote care for inpatient care can be developed to (1) enhance visibility affordances of digital technologies to provide, for example, spatial perception and an integration of segregated views with the monitoring devices and (2) advance a balanced model of virtual and physical forms of visibility.

Integrating the design of the built environment with the design of the technologies for service delivery, and balancing physical and virtual forms of visibility, can help support future transformations of the healthcare ecosystem involving the integration of hospital care with home and community care.

While this case study is based on an innovative solution for inpatient telemedicine, further work is needed to compare and evaluate different solutions and implementations of telemedicine technologies for COVID-19 ICUs. More studies on the impact of virtual visibility versus physical visibility on the performance of hospital units are needed as well as studies on the implementation of inpatient telemedicine in different medical specialties and models of care. Further research on the impact of inpatient telemedicine on the design of healthcare facilities in diverse environmental, cultural, and economic contexts will enhance the knowledge base needed for the future development of healthcare architecture and digital technologies for remote care.

## Implications for Practice

- Different from telemedicine for outpatient care, where virtual visibility is mainly a means for enhanced communication between the caregiver and the patient, in inpatient care, and specifically in ICU, virtual visibility needs to support different purposes including management of the unit, teamwork between caregiver teams, and remote monitoring of patients by caregivers in changing conditions over time.
- The case study demonstrated the limitations of current telemedicine technologies that were not designed for inpatient care to account for the holistic conception and spatial integration of the unit and the dynamic use of the space that are crucial for orientation, monitoring, control, and collaboration.
- The combination of physical visibility based on the user’s dynamic perception while present in space, or on-site, and virtual visibility that provides remote control over distance, movement, and angle of view was significant to the performance of the unit.
- The case study showed that the use of digital technologies for inpatient telemedicine had unintended and unexpected uses for infection control, family support, or human augmentation.
- The case study presents the potential of a hybrid model of virtual and physical forms of visibility that provides diverse patterns of visibility to enhance safety, quality of care, and well-being.

## Supplemental Material

Supplemental Material, sj-docx-1-her-10.1177_19375867211009225 - Telemedicine Implementation in COVID-19 ICU: Balancing Physical and Virtual Forms of Visibility Click here for additional data file. Supplemental Material, sj-docx-1-her-10.1177_19375867211009225 for Telemedicine Implementation in COVID-19 ICU: Balancing Physical and Virtual Forms of Visibility by Nirit Putievsky Pilosof, Michael Barrett, Eivor Oborn, Galia Barkai, Itai M. Pessach and Eyal Zimlichman in HERD: Health Environments Research & Design Journal
