# Supplementary material for: Telemedicine Implementation in COVID-19 ICU: Balancing Physical and Virtual Forms of Visibility
Source: HERD. 2021 Jun 2;14(3):34–48. doi: 10.1177/19375867211009225 (PMC8212392; doi:10.1177/19375867211009225)
Supplement: Supplemental Material, sj-docx-1-her-10.1177_19375867211009225 - Telemedicine Implementation in COVID-19 ICU: Balancing Physical and Virtual Forms of Visibility [file sj-docx-1-her-10.1177_19375867211009225.docx]

Appendix 1

Appendix 1. List of sources for the research

| **Interviews (30 total)** |
| --- |
| 11 physicians  3 nurses  3 IT administrators  2 Human experience  5 Startup directors  6 Architects & engineers |
| **On-site visits during June-Aug 2020** |
| Guided tours of the COVID units (2).  Observations in the control room (2 days).  Presentation & discussion with the hospital board of directors. |
| **Documentation** |
| Sheba MC brief of the telemedicine technologies implemented in the COVID units.  Telemedicine startups documents.  Architectural drawings.  TV Documentaries.  Media publications.  Webinars of Sheba Medical Center and the ARC Innovation center. |
